# Bacterial contamination in contact lens training area in private optical clinics

**DOI:** 10.1186/s12348-024-00407-z

**Published:** 2024-06-11

**Authors:** Sana Badar Baig, Kalaivani Manokaran, Nagarajan Theruveethi, Vivek Raghavan Muduthan

**Affiliations:** 1 Department of Medical Laboratory Technology, Manipal College of Health Professions, Manipal Academy of Higher Education, Manipal, Karnataka 576104 India; 2 Department of Optometry, Manipal College of Health Professions, Manipal Academy of Higher Education, Manipal, Karnataka 576104 India

**Keywords:** Bacterial contamination, Contact lens hygiene, Contact lens complications, Private eye clinic, Contact lens container, Solution

## Abstract

**Background:**

Contamination in the contact lens training area could be due to bacteria, which can lead to the major consequence of ocular infections. We aimed to investigate the contamination caused by bacteria in the contact lens training area in private optical clinics of the Udupi district, India.

**Methods:**

A cross-sectional study evaluated the swabs from the contact lens container, contact lens solution tip, washing area and lens fitting area for bacterial contamination. Twenty swabs collected from different areas of five optical clinics were inoculated in Brain heart infusion broth (BHIB). The broth was streaked in MacConkey and Blood agar and incubated at standard conditions for the growth of bacteria. All isolates were identified using conventional culture methods, and Gram staining was performed.

**Results:**

Twenty samples (contact lens case, *n* = 5; contact lens solution tip, *n* = 5; washing area, *n* = 5; cleaning towel, *n* = 5) from private optical clinics were recruited for the study. Bacterial growth was indicated in which lactose fermentation was seen at (15%), non-lactose fermentation at (35%), and no bacterial growth at (50%) in MacConkey agar. Partial or alpha-hemolytic (α hemolysis) was seen in (5%), complete or beta-hemolytic (β hemolysis) was seen in (40%), no hemolysis or gamma hemolysis (ϫ haemolysis), was seen in (30%), no growth was seen in (25%) on blood agar. Gram-positive cocci (45%), Gram-negative bacilli (20%), and no increase in (35%) were observed in MacConkey agar and Blood agar. Bacterial species were not identified in this study.

**Conclusion:**

Contamination was found in lenses, solution tips, washing areas, and cleaning towels which might lead to ocular infections. Perception should be given to those responsible for fitting lenses.

**Supplementary Information:**

The online version contains supplementary material available at 10.1186/s12348-024-00407-z.

## Introduction

Vision improvement technology via contact lenses in close contact with the cornea was initially practised. The commercial contact lens was first manufactured and introduced by Otto Wichterle, who endlessly transformed the contact lens industry with his contributions to soft hydrogels [1–3]. Contact lenses are progressively widespread; it has been estimated that about 125 million individuals worldwide wear contact lenses for cosmetic purposes like the adaptation of eye colour as well as conventional prosthetic devices, which includes therapeutic reasons to reverse vision correction because it is lightweight and virtually invisible [4, 5]. Soft contact lenses are prone to attract bacteria and many other pathogens, and they have increased chances of transmitting from the lens to the corneal epithelium, potentially leading to an eye infection [5]. In accumulation to these complications, abundant studies have revealed that contact lenses, contact lens cases and contact lens care solutions were usually burdened with up to 10^7^ colony-forming units (CFU) of bacteria [6, 7]. Mainstream contact lens users are unfamiliar and oblivious to their care practices and contact lens wear complications [8, 9]. Due to improper hygiene in handling contact lenses and their accessories in numerous cases, contact lens users cause contamination themselves [10]. Microbial contamination in contact lens fitting can be related to corneal infection and inflammation. Studies have explored quantifying the contamination of contact lens cases and solutions [5–7, 10]. The contaminations in contact lenses have been examined, but studies have not been conducted to inspect the bacterial contamination in the contact lens area of private optical clinics. The current study aims to identify the bacteriological contamination in the contact lens training area to know whether contact lenses and their accessories transmit bacterial contamination.

## Materials and methods

This cross-sectional study was conducted in the Department of Medical Laboratory Technology, Manipal. A total of 5 private optical clinics in Udupi district, Karnataka, India, with lens care fitting facilities were selected for the study. The study was conducted in accordance with the Declaration of Helsinki after obtaining approval from the Institutional Research Committee and Institutional Ethics Committee; IEC: 220/ 2021. Written informed consent was attained, and a participant information sheet, which included all the necessary information regarding the research, was provided to optical authorities before the data collection.

### Sample collection processing

The author (SB) prepared the agar medium before sample collection, and the optical was selected randomly, and the optical authorities were not instructed beforehand to avoid bias. A total number of 5 optical clinics of Udupi district were sampled in which 4 different swabs were collected from areas of the optical clinic, which included contact lens case, contact lens solution tip, washing area and cleaning towel, were microbiologically examined for turbidity, colony formation in MacConkey agar and Blood agar and morphology through Gram staining.

### Sample Transportation

 The collected samples were sealed and labelled with information including participant identification code, area of swab collected and time of collection. The swabs (HY-LiTE® Hygiene, Merck KGaA, Darmstadt, Germany) were transported using CryoPod Carrier Portable Sample Transport from (Capacity: 1 × 2″ Cryobox) (Azenta Life Sciences 101; Witmer Road, Suite 700; Horsham, PA 19,044) to the laboratory for microbiological examination within 1 h of collection based on standard operating procedures and aseptic in a sterile container. To avoid contamination with indigenous flora, we followed typical aseptic. We made sure to target only one optical per day to avoid delays in sample transportation.

### Culture and bacterial growth

The swabs were inoculated in Brain Heart Infusion Broth (BHIB) (HiMedia Laboratories, India) and incubated at 37 °C for 24 h. After incubation, the broths were examined for turbidity, which indicates growth. After 24 h, a loop full of culture broth was sub-cultured by the streaking method (four-quadrant method) in Blood agar (HiMedia Laboratories, India) and MacConkey agar (HiMedia Laboratories, India). Inoculated plates were incubated at 37 °C for 18–24 h. After 24 h, colony morphology was identified using the Gram-staining method [7].

### Microbiological examination

We used McFarland [11] (“A 0.5 McFarland standard is prepared by mixing 0.05 mL of 1.175% barium chloride dihydrate (BaCl_2_•2H_2_O), with 9.95 mL of 1% sulfuric acid (H_2_SO_4_)”) standard criteria to interpret turbidity as an indicator of bacterial growth which was infused in BHIB [12].

## Results

**Fig. 1:**
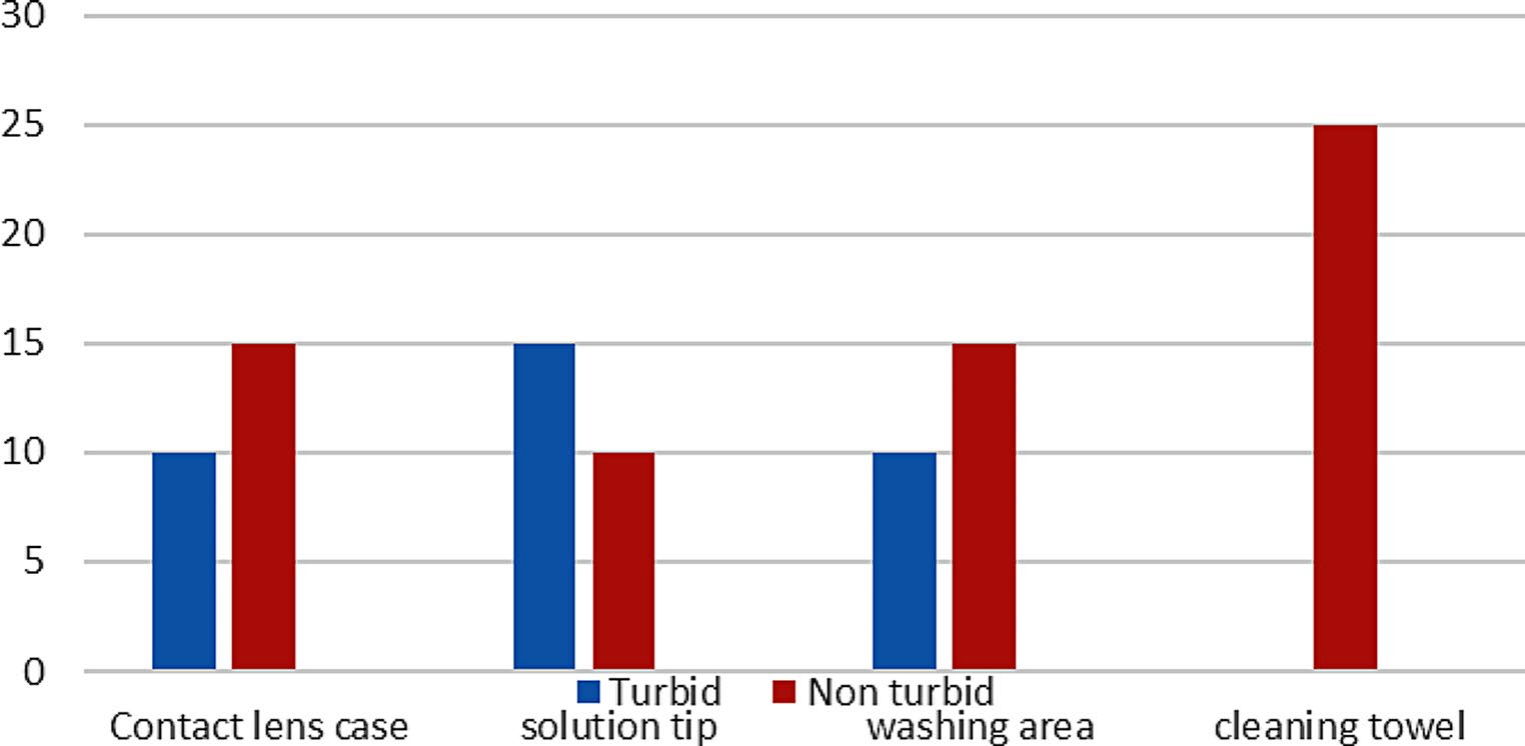
Demonstrates the percentage of growth in BHIB from various contact lens fitting area sites

The bacteria were grown to produce out of 20 samples collected from 5 private optical clinics with lens training areas in the Udupi district; turbidity was noted in 7 samples, and 13 samples were non-turbid. The five contact lens case samples collected from different private optics showed 10% turbidity and 15% non-turbidity,5 samples of contact lens solution tip showed 15% turbidity and 10% non-turbidity, five samples of washing area showed 10% turbidity and 15% were non-turbidity,5 samples of cleaning towel showed 25% non-turbidity (Fig. 1). From 20 samples, 35% samples indicated growth and 65% stated that there was no growth based on turbidity in BHIB.

### Cultural characteristics of bacterial colonies in MacConkey agar

A total of 20 samples were collected and analyzed, of which 3 were found to be lactose fermenting, 7 were non-lactose fermenting, and 10 showed no growth. On MacConkey agar, it was noted that no growth was observed in 5 samples collected from contact lens cases. In comparison, 1 sample showed a non-lactose fermenting colony in MacConkey agar from 5 samples collected from the contact lens solution tip. In addition, 3 samples indicated lactose fermenting colonies and 2 samples indicated non-lactose fermenting colonies in MacConkey agar from 5 samples collected from the washing area, and 1 sample indicated lactose fermenting colonies, and 4 samples indicated non-lactose fermenting colonies in MacConkey agar from 5 samples collected from the cleaning towel. The results showed that 15% of the samples were lactose fermenting, 35% were non-lactose fermenting, and 50% showed no growth on MacConkey agar.

### Hemolysis characteristics of bacteria in blood agar

Out of 20 samples collected from private optical clinics, 1 sample showed partial or alpha-hemolytic (α hemolysis), 8 samples showed complete or beta-hemolytic (β hemolysis), 6 samples showed no hemolysis or gamma hemolysis (ϫ hemolysis). 5 samples were non-hemolytic. The 5 samples collected from the contact lens case, 3 showed complete or beta-hemolytic (β hemolysis), and 1 was non-hemolytic or gamma hemolysis (ϫ hemolysis). Out of 5 samples collected from the solution tip, 2 samples showed complete or beta-hemolytic (β hemolysis), and 1 was non-hemolytic or gamma hemolysis (ϫ hemolysis) out of 5 samples collected from the washing area, 1 sample was partial or alpha-hemolytic (α hemolysis),2 samples showed complete or beta-hemolytic (β hemolysis), and 1 was non-hemolytic or gamma hemolysis (ϫ hemolysis), Out of 5 samples collected from cleaning towel 2 samples showed complete or beta-hemolytic (β hemolysis), and 3 samples were non-hemolytic or gamma hemolysis (ϫ hemolysis). 5% of samples were partial or alpha-hemolytic (α hemolysis), 40% of samples showed complete or beta-hemolytic (β hemolysis), 30% of samples showed no hemolysis or gamma hemolysis (ϫ hemolysis), 25% showed no growth.

### Colony morphology in positive culture cases

**Fig. 2:**
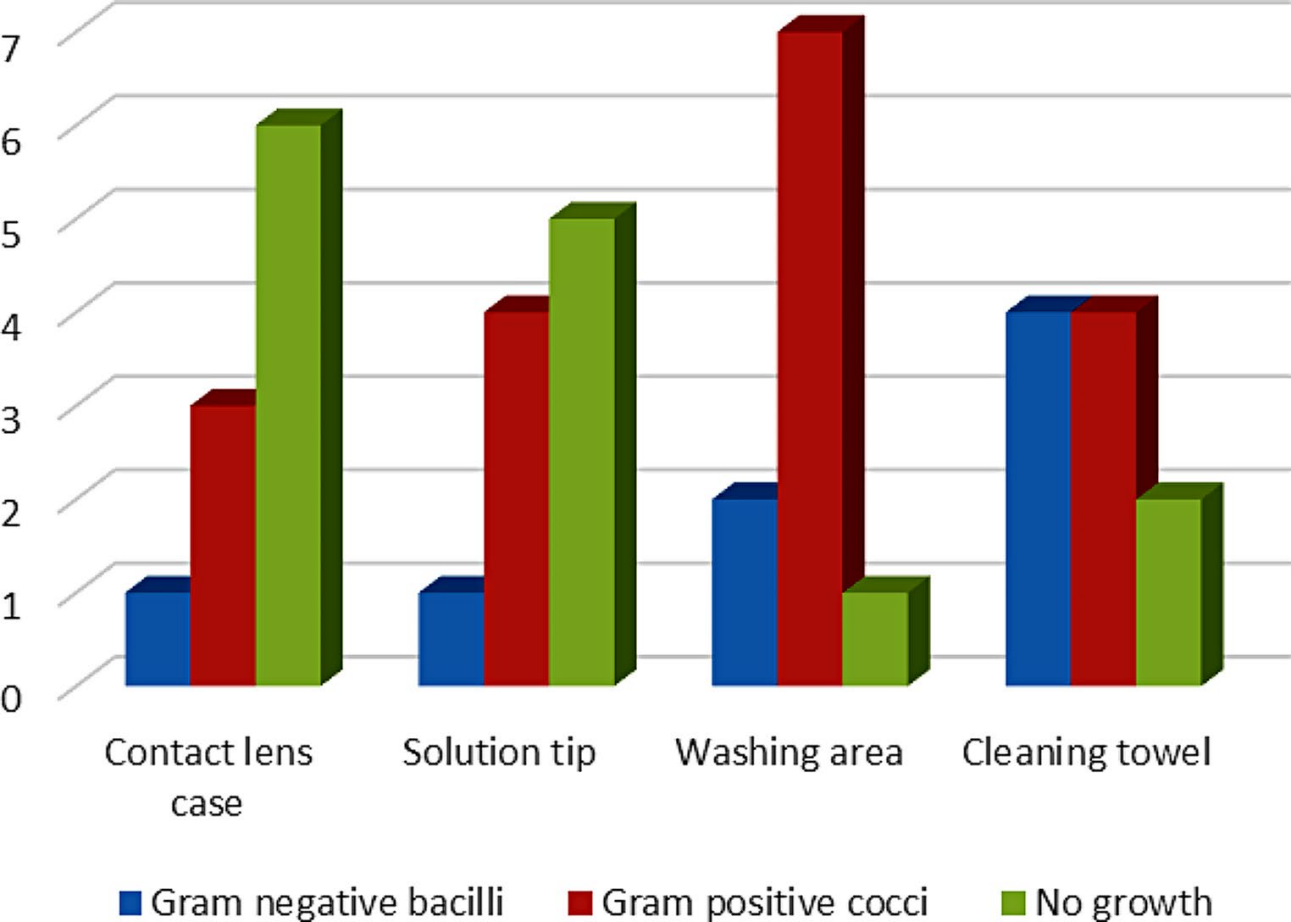
Total number of Gram-positive and Gram-negative bacteria from samples collected from various parts of the lens fitting area

Out of 20 samples in MacConkey agar, Gram-positive cocci were observed in 6 samples, Gram-negative bacilli were observed in 3 samples, and 11 showed no growth. Out of 20 samples, blood agar and gram-positive cocci were observed in 12 samples, gram-negative bacilli were observed in 5 samples, and 3 samples showed no growth. 1 Gram-negative bacilli, 3 Gram-positive cocci were observed in samples collected from contact lens cases, 1 Gram-negative bacilli, 4 Gram-positive cocci were observed in samples collected from solution tip, 2 Gram-negative bacilli, 7 Gram-positive cocci were observed in samples collected from washing area, 4 Gram-negative bacilli, 4 Gram-positive cocci were observed in samples collected from a cleaning towel. Out of 20 MacConkey agar and 20 blood agar samples, 45% were gram-positive cocci, 20% were gram-negative bacilli, and the remaining 35% showed no growth (Fig. 2).

## Discussion

To retain harmless contact lens wear, contact lenses, and other contact lens-related fittings including the lens storing cases, contact lens case contamination has been recognised as one of the primary sources of contact lens contamination. The lens case may act as a reservoir to transfer the microbes to lenses and then to the eye. Contact lens storage cases are frequently contaminated—the microorganisms causing microbial keratitis have been traced in affected individuals [13].

We found that bacterial contamination in 35% of samples indicated growth and 65% indicated no growth based on turbidity in BHIB. The result concorded with a previous study in corneal scrape profiles indicated bacterial growth and found bacteria causing microbial keratitis in BHIB isolated samples [14]. A similar study found that out of 100 contact lens wearers, 65% of contact lenses, 89% of lens storage cases and 32% of conjunctiva were contaminated. Contamination in the contact lens care system is the chief cause of contact lens wear infections [15]. We found lactose fermentation in 15% of samples, 35% were non-lactose fermenting, and 50% showed no growth on MacConkey agar. Lactose fermentation in contact lenses decreases the pH [16], eventually leading to corneal acidosis, which can trigger corneal hypoxia, especially to epithelia, which reduces the stromal pH triggering corneal oedema and infiltrates [16–18]. The Blood agar showed partial hemolysis in 5% of samples, whereas 40% showed complete hemolysis, 30% showed no hemolysis, and 25% showed no growth. This can be a phenotypic bacterial pathogen, especially streptococcus [19], which could cause keratitis and epithelial defects [20]. Thakur et al. backed this, showing 52% of bacterial growth in 200 samples. Out of these, 62% of lens cases were contaminated the most contaminated, followed by 56% lenses, 48% tip of solution bottles, and 42% lens care solution. It also indicated that the lens care practices among the research participants were not the finest, which resulted in the elevation of contamination and suggested increasing the attention towards hygiene. In contrast, lens application and frequent cleaning and replacement of lens cases and solutions should be practised [21]. Our study also presented bacterial growth in samples collected from the washing area and cleaning towels, which may also play a crucial part in the contamination of contact lenses.

Current study results showed 8 and 18 positive cultures from Blood and MacConkey agar, of which 69% were Gram-positive cocci and 31% were Gram-negative bacilli. The study conducted by Ananya showed 42% of contaminated lens cases with Gram-positive bacteria only, 24% of contaminated lens cases with Gram-negative bacteria and 35% of lens cases containing Gram-positive and Gram-negative bacteria [13]. A review-based study found that contact lens kits usually contain 10% gram-negative bacteria and other infection-causing bacterial species. This study concluded that microbial keratitis associated with contact lenses may be caused by the existence or transmission of microorganisms from the lens to the eye surface. Accurate knowledge of lens-associated contamination is vital to inhibit the colonisation of bacteria on contact lenses and contact lens cases, which may reduce the rate of infection caused by bacteria [21]. Our study could not identify the bacterial species causing contamination and the microbial load in the lens fitting area of private optical clinics. This study has a few limitations as we did not include an evaluation of the hygiene practice carried out in the contact lens fitting area of private optical clinics, did not assess the risk of infection, and instead only evaluated the presence of bacteria or absence of bacteria that may not accurately point of the ocular infection related to contact lens hygiene. The sample size was not powered to estimate the incidence of bacterial contamination. Further investigation isolates the types of bacteria in the training area that can lead to contact lens-related infections, which could help to comprehend the practice.

## Conclusion

Maintaining good hygiene by frequently washing hands and sanitising the area can significantly reduce the risk of infection and lens dropouts. It is essential to be mindful of bacterial contamination in the contact lens fitting area, as it can lead to contact lens-related bacterial infection. The appropriate awareness should be provided to those responsible for executing the lens fitting technique (Eye care practitioners, especially in private optical setups), as they will likely encounter pathogenic bacteria that can easily twig to the contact lens.

### Electronic supplementary material

Below is the link to the electronic supplementary material.

Supplementary Material 1

## Data Availability

The data is reported in the study available with corresponding authors it will be shared upon reasonable request.
