# Supplementary material for: Bacterial contamination in contact lens training area in private optical clinics
Source: J Ophthalmic Inflamm Infect. 2024 Jun 11;14:26. doi: 10.1186/s12348-024-00407-z (PMC11166607; doi:10.1186/s12348-024-00407-z)
Supplement: Supplementary file 1 — Supplementary Material 1 [file 12348_2024_407_MOESM1_ESM.docx]

**Table 1: Number of turbid and non-turbid samples in BHIB**

| **Growth in BHIB** | **No. of samples** | **Percentage** |
| --- | --- | --- |
| Turbid | 7 | 35% |
| Non-turbid | 13 | 65% |
| Total | 20 | 100% |

**Table 2: Total number of Lactose and Non-lactose fermenting colonies on MacConkey agar**

| **Growth on MacConkey agar** | **Total number** | **Percentage** |
| --- | --- | --- |
| Lactose fermenting | 3 | 15% |
| Non lactose fermenting | 7 | 35% |
| No growth | 10 | 50% |
| Total | 20 | 100% |

**Table 3: Cultural characteristic in MacConkey and Blood agar from samples collected from contact lens case**

| **Contact lens case** | **MacConkey agar** | **Blood agar** |
| --- | --- | --- |
| **Participant 1** | **No growth** | **Pinhead, circular, glistering, entire, raised, mucoid, opaque, non-hemolytic** |
| **Participant 2** | **No growth** | **1. Small, round, moist.**  **2.pinhead, round, moist, beta-hemolytic** |
| **Participant 3** | **No growth** | Medium, irregular, moist beta-hemolytic |
| **Participant 4** | **No growth** | **Medium, irregular, moist, beta-hemolytic** |
| **Participant 5** | **No growth** | **No growth** |

**Table 4: Cultural characteristic in MacConkey and Blood agar from samples collected from solution tip**

| **Contact lens solution tip** | **MacConkey agar** | **Blood agar** |
| --- | --- | --- |
| **Participant 1** | No growth | No growth |
| **Participant2** | No growth | Small, round ,dry , non-hemolytic |
| **Participant3** | Small, round, non-lactose fermenting | Medium, irregular, moist, Beta-hemolytic |
| **Participant 4** | No growth | Small, greyish, round, beta-hemolytic |
| **Participant 5** | No growth | No growth |

**Table 5: Cultural characteristic on MacConkey and Blood agar from samples collected from washing area**

| **Washing area** | **MacConkey agar** | **Blood agar** |
| --- | --- | --- |
| **Participant 1** | **Round, non-lactose fermenting** | **No growth** |
| **Participant 2** | Round, mucoid, lactose fermenting | Round, large greyish, irregular beta-hemolytic |
| **Participant 3** | Round, shiny, moist, lactose fermenting | Irregular, moist, non-hemolytic |
| **Participant 4** | Small, round, moist, non-lactose fermenting | Medium, moist, irregular, Alpha-hemolytic |
| **Participant 5** | Small, round, mucoid, Lactose fermenting | Round, small, whitish, beta haemolytic |

**Table 6: Cultural characteristic on MacConkey and Blood agar from samples collected from cleaning towel**

| **Cleaning towel** | **MacConkey agar** | **Blood agar** |
| --- | --- | --- |
| **Participant 1** | **No growth** | **Small, round, white, non-hemolytic** |
| **Participant 2** | Small, round, moist, non -lactose fermenting | Whiteish, small round ,moist, beta haemolytic |
| **Participant 3** | Pin point, scanty growth, moist, non-lactose fermenting | Small, round, moist, non-hemolytic |
| **Participant 4** | Small, round, moist, non-lactose fermenting | Whiteish, small moist, round, beta-hemolytic small, round, moist, non-hemolytic |
| **Participant 5** | Small, moist, non-lactose fermenting | No growth |

**Table 7: Number of alpha, beta and gamma hemolysis on Blood agar**

| **Haemolysis** | **Number** | **Percentage** |
| --- | --- | --- |
| Alpha hemolytic | 1 | 5% |
| Beta hemolytic | 8 | 40% |
| Gamma hemolytic | 6 | 30% |
| Non hemolytic | 5 | 25% |
| Total | 20 | 100% |

**Table 8: Total number of samples demonstrating Gram-positive and Gram-negative bacteria on MacConkey and Blood agar**

| **Gram stain** | **MacConkey agar** | **Blood agar** | **Percentage on Blood and MacConkey agar** |
| --- | --- | --- | --- |
| Gram Positive cocci | 6 | 12 | 45% |
| Gram negative bacilli | 3 | 5 | 20% |
| No growth | 11 | 3 | 35% |
| Total | 20 | 20 | 100% |

**Table 9: Sample collected from contact lens cases**

| **Contact lens case** | **Gram stain in MacConkey** | **Gram stain in blood agar** |
| --- | --- | --- |
| **Participant 1** | **No growth** | **Gram-negative bacilli** |
| **Participant 2** | **No growth** | **Gram-positive cocci** |
| **Participant 3** | **No growth** | **Gram-positive cocci** |
| **Participant 4** | **No growth** | **Gram-positive cocci** |
| **Participant 5** | **No growth** | **No growth** |

**Table 10: Sample collected from solution tip**

| **Solution tip** | **Gram stain in MacConkey** | **Gram stain in blood agar** |
| --- | --- | --- |
| **Participant 1** | **No growth** | **No growth** |
| **Participant 2** | **No growth** | **Gram-negative bacilli** |
| **Participant 3** | **Gram-positive cocci** | **Gram-positive cocci** |
| **Participant 4** | **Gram-positive cocci** | **Gram-positive cocci** |
| **Participant 5** | **No growth** | **No growth** |

**Table 11: Sample collected from washing area**

| **Washing area** | **Gram stain in MacConkey** | **Gram stain in blood agar** |
| --- | --- | --- |
| **Participant 1** | **No growth** | **Gram-positive cocci** |
| **Participant 2** | **Gram-positive cocci** | **Gram-positive cocci** |
| **Participant 3** | **Gram-positive cocci** | **Gram-positive cocci** |
| **Participant 4** | **Gram-negative bacilli** | **Gram-negative bacilli** |
| **Participant 5** | **Gram-positive cocci** | **Gram-positive cocci** |

**Table 12: Sample collected from cleaning towel**

| **Cleaning towel** | **Gram stain in MacConkey** | **Gram stain in blood agar** |
| --- | --- | --- |
| **Participant 1** | **No growth** | **Gram-positive cocci** |
| **Participant 2** | **Gram-negative bacilli** | **Gram-negative bacilli** |
| **Participant 3** | **Gram-negative bacilli** | **Gram-negative bacilli** |
| **Participant 4** | **Gram-positive cocci** | **Gram-positive cocci** |
| **Participant 5** | **No growth** | **Gram-positive cocci** |
